# On the Conservation of the Canarian Laurel Forest: What Do Lichens Have to Say?

**DOI:** 10.3390/jof10100668

**Published:** 2024-09-25

**Authors:** Cristina González-Montelongo, Israel Pérez-Vargas

**Affiliations:** Department of Botany, Ecology and Plant Physiology, Faculty of Pharmacy, University of La Laguna Apdo, P.O. Box 456, 38200 La Laguna, Tenerife, Canary Islands, Spain; cgonzalm@ull.edu.es

**Keywords:** laurel forest, lichens, extinction debt, functional traits, Canary Islands, Macaronesia

## Abstract

The fragmentation and degradation of primary forests are serious threats to the long-term persistence not only of the tree species they comprise, but also of many organisms inhabiting them. The Canarian laurel forest, known as monteverde, is a highly threatened endemic forest of the Macaronesian region. Lichens are considered ideal bioindicators for assessing the effects of human disturbances on ecosystems and anticipating the response of other less sensitive organisms. However, no studies have used them as model organisms to analyze the conservation status of this primary forest in the Macaronesian region. In the present study, we analyzed several variables of the lichen biota of the Canarian laurel forest on the islands with the highest representation within this archipelago: La Gomera, La Palma, and Tenerife. We analyzed the species richness (and its relationship to island size with the real and potential vegetation areas of the laurel forest); the lichen diversity value, the number of shared and exclusive species on each of the islands as well as lichen functional traits as they have become important for evaluating the response of epiphytic lichens to environmental changes. The results indicate that there are signs of a potential extinction debt occurring in the diversity of epiphytic lichens in some areas. Furthermore, it has been observed that, despite the presence of some exclusive species on each island, the overall composition does not differ between them. Considering the functional traits of lichens, there are patterns that can provide information about the unique characteristics of the laurel forest of each of the studied islands.

## 1. Introduction

Earth’s biodiversity encompasses millions of species, many of which remain undiscovered or undescribed by science. Islands, despite harboring a disproportionate amount of Earth’s biodiversity compared to continents, are particularly vulnerable to anthropogenic disturbances [1]. The often highly restricted range of distribution and small population size of insular species, together with their limited diversity of defenses, make island biotas particularly vulnerable to extinction [2,3].

The fragmentation of habitats has emerged as a significant conservation concern due to its adverse effects on the decline and extinction of plant species. Nonetheless, the local extinction of species might transpire with a temporal delay subsequent to ecosystem perturbation, a phenomenon known as extinction debt [4,5]. Quantifying such extinctions and investigating the long-term consequences of perturbations have proven challenging because these perturbations are not isolated and occur across various spatial and temporal scales, from local habitat losses to global warming [6].

For over 150 years, oceanic or volcanic islands have been the subject of numerous studies that have led to the development of various theories on biodiversity, biogeography, ecology, etc. [7,8,9,10,11]. The Macaronesian archipelagos have not been an exception, and there are several examples in this regard (see [12,13,14,15]). However, not all biological groups have been approached with the same intensity. In the specific case of lichens, there are numerous works on their diversity in the islands, and new records and species continue to appear with some frequency [16,17,18,19]. Laurel forests are forest ecosystems that present the best conditions for the development of a higher diversity and abundance of lichens. The Macaronesian laurel forests, also known as laurisilva or monteverde, are lush evergreen forests found within the cloud belt of the Macaronesian islands [20]. These forests are characterized by their dominance of evergreen broadleaf laurifolious trees, making them the most diverse forest ecosystems in the islands. The tree canopy consists of approximately twenty different tree species [21]. Moreover, these forests boast a remarkable richness and diversity of cryptogamic life, particularly lichens [22,23,24]. Designated as a priority habitat under Annex I of the Habitats Directive, laurel forests hold significant importance in the Macaronesian region due to their exceptionally high levels of endemic biodiversity [25]. Human activity has been modifying the native forests in Macaronesia for at least five centuries. As a result of extensive exploitation, for instance, in the Canary Islands, only 11.8% of the original laurel forests remain in this archipelago today [21,26]. Cloud forests are regarded as one of the most vulnerable terrestrial ecosystems [27]. Several approaches have been made to understand the conservation status of this forest from the perspective of fauna and flora [20,21,28,29]. Despite their significance, both in terms of species diversity and their established capacity to detect environmental changes, lichens have not been utilized in the Canary Islands, or even in the Macaronesian region, to assess the conservation status of endemic forests. Furthermore, there is a notable absence of research concerning the extinction risk of lichens within our region under the current landscape configurations and management practices as well as the additional impacts of climate change [30]. This study aims to address this gap by evaluating the conservation status of the Canarian laurel forest through its epiphytic lichen biota. To achieve this, we examined various factors including species richness, lichen diversity indices, and functional traits of lichens.

## 2. Materials and Methods

### 2.1. Study Area

This study was carried out in the Canary Islands. Nowadays, the La Palma, La Gomera, and Tenerife Islands have the best preserved and most extensive laurel forests in the Canary Islands. For this reason, the present study was conducted on these three islands.

**Figure 1:**
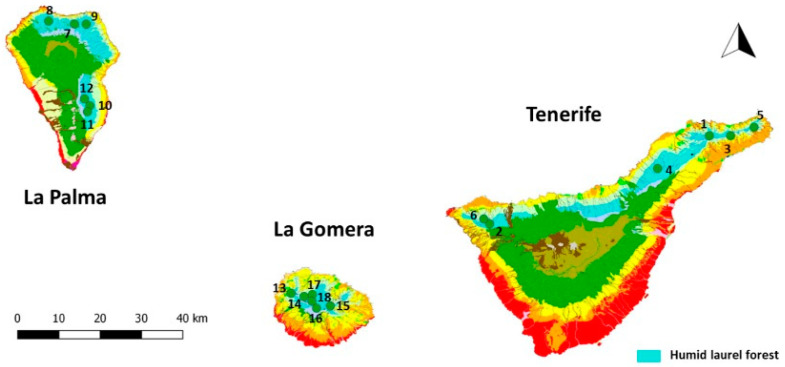
Field plots installed in a potential area of humid laurel forest (*Lauro novocanariensis—Perseetum indicae*) on the La Palma, La Gomera, and Tenerife Islands.

The Macaronesian cloud forests constitute an environmentally complex system characterized by different plant associations of evergreen laurel forests. In order to mitigate the impact of these variations on lichen communities and to standardize our sampling sites as much as possible, all plots were consistently situated within the potential distribution area of a humid laurel forest (*Lauro novocanariensis-Perseetum indicae*; Figure 1) featuring homogeneous mesoclimatic conditions: mild temperatures year-round, absence of freezing or snowfall in winters, mean annual temperatures ranging between 13 and 18 °C, and annual rainfall between 500 and 1200 mm with a dry season in summer, albeit influenced by prevailing NE trade winds that induce frequent low-level cloud cover and additional water supply from fog, primarily occurring in the early summer and early autumn [21,31]. Fieldwork was carried out on these islands, between 750 and 1100 m a.s.l., where the humid laurel forest grows (Table 1).

### 2.2. Plot Selection and Sampling

Our field campaigns were authorized by *Área de Medioambiente-Cabildo Insular de Tenerife*, *Área de Medioambiente-Cabildo Insular de La Palma*, and by *Parque Nacional de Garajonay*, with permission to access and collect data and biological material.

Six sampling plots of 10 m × 10 m were designated on each island. Within each plot, eight trees were chosen, consisting of two individuals from each of the four most prevalent tree species in this forest: (i) *Morella faya* (Aiton) Wilbur, (ii) *Erica canariensis* Rivas-Mart., M. Osorio & Wildpret, (iii) *Laurus novocanariensis* Rivas-Mart., Lousa, Fern. Prieto, E. Días, J.C. Costa & C. Aguiar, and (iv) *Ilex canariensis* Poir. *in* Lamarck. The selection of these four tree species was not solely based on their diverse bark properties, leaf characteristics, architecture, and canopy structure, but also because of their abundance and common occurrence in the laurel forest, justifying their inclusion in each designated plot.

Only living, corticated trees with a stem diameter at breast height (DBH) > 22 cm were included in the sampling. Lichen samples were collected following the protocol outlined by [32,33], with some modifications (see [24]). A frame measuring 50 cm × 10 cm, subdivided into five quadrats of 10 cm × 10 cm each, was employed for sampling. The uppermost edge of the frame was positioned at 1.5 m above ground level, and adjusted up to a maximum of 2 m if the trunk was unsuitable at the desired height. The survey was conducted between September 2013 and March 2018.

### 2.3. Specimen Identification

In each microplot, all lichens (both macro and microlichens) were removed and subsequently identified in the laboratory. The names and authors of the taxa are related in the Supplementary Materials (Table S1). The names of the authors were omitted from the text, and the nomenclature of the lichen species mainly followed [34]. The morphology of the lichen specimens was examined using a Leica Zoom 2000 microscope (Buffalo, NY, USA). Sections for anatomical analysis were hand-cut and studied under an Olympus CH compound microscope (Shinjuku, Japan). Ascospore measurements were conducted in water at 1000× magnification, with only well-developed spores located outside the asci being measured. In some cases, the identification of chemical constituents (secondary metabolites) was necessary for accurate sample identification. Thin-layer chromatography (TLC) was utilized following standard procedures [35,36]. Voucher specimens were deposited in the TFC-Lich Herbarium at the University of La Laguna (Spain).

### 2.4. Lichen Functional Traits

Species were classified in groups according to the following traits. (1) Photobiont: green algae, *Trentepohliaceae*, and cyanobacteria, (2) growth form: leprose, crustose, squamulose, foliose, fruticose, and cladoniiform thallus, and (3) reproductive strategy: asexual (thallus fragmentation, isidia, soralia, isidia + soralia) and sexual (spores). Furthermore, the species were categorized into functional groups based on their ecological requirements (tolerance to eutrophication, water requirements, solar irradiation, poleotolerance, and pH of the substrata) according [37,38] (Table 2).

In cases where ecological data were not available in the literature for certain species, the values for these ecological indicators were assigned based on expert assessments by Canarian lichenologists and our own field observations.

### 2.5. Data Analysis

To examine lichen diversity, we calculated the species richness (S), and lichen diversity values (LDVs) in each plot [32,33]. Differences in S and LDV were assessed using the medians of the Kruskal–Wallis nonparametric test (H; *p* ≤ 0.05).

We represented exclusive vs. shared species among islands using a Venn diagram, created using the online tool Venny 2.1.0 [39].

The dependence relationship between lichen functional groups and islands was studied with the chi-square test. The ecological requirements of lichens (pH, solar irradiation, aridity, eutrophication, and poleotolerance) were studied with the community-weighted mean (CWM) [40]. We built an abundance matrix with plot, island, and exposure following the method in [41]. We computed nonmetric multidimensional scaling (NMDS) analyses with 200 permutations to elucidate the level of similarity among plots in a geometric space with few dimensions. In the NMDS analyses, we utilized the Jaccard distance, given its effectiveness as one of the primary measures for community data analysis [42]. To study the adequacy of the sample configuration in the NMDS, we analyzed the stress value [43]. Finally, to ascertain the presence of significant differences between islands, an ANOSIM test was conducted [44]. Additionally, to identify the lichen species exerting the greatest influence on the similarity (or dissimilarity) between islands, a SIMPER analysis was employed [44].

The S, LDV, Kruskal–Wallis, chi-square test, and box-plot were analyzed and represented using Paleontological Statistics (PAST) v.3.12 [45]. The CWM representations and dispersion graph were created in Microsoft^®^ Excel (Office^®^ 2021). Finally, NMDS, ANOSIM, and SIMPER analyses were conducted and visualized using CAP (Community Analysis Package 3.11) [46].

Following [12], we investigated the island species–area relationship (ISAR) by examining the correlation between the total number of observed species on each island and its respective total area, potential area of humid laurel forest distribution, and current distribution area of humid laurel forest by utilizing data provided by [21]. Finally, species richness (S) was plotted against the aforementioned areas using a log/log graph by calculating the linear regression line, its explicit equation, and its coefficient of determination (R^2^). This representation was made using the Microsoft Excel^®^ program.

## 3. Results

### 3.1. Lichen Diversity

A total of 165 epiphytic lichens species (grouped in 34 families and 71 genera) were identified (Table S1; Supplementary Materials). By island, Tenerife is where the most species were found, with 96 taxa, followed by La Palma with 82, and finally La Gomera with 70. The five most frequent families in the laurel forests of the three studied islands were *Parmeliaceae* (32 taxa), *Ramalinaceae* (15), *Lobariaceae* (12), *Lecanoraceae* (11), and *Pertusariaceae* (10), and the five most frequent species were *Parmotrema perlatum* (202 samples), *Chrysothrix candelaris* (142), *Leucodermia leucomelos* (117), *Phlyctis agelaea* (84), and *Bacidia absistens* (73).

**Figure 2:**
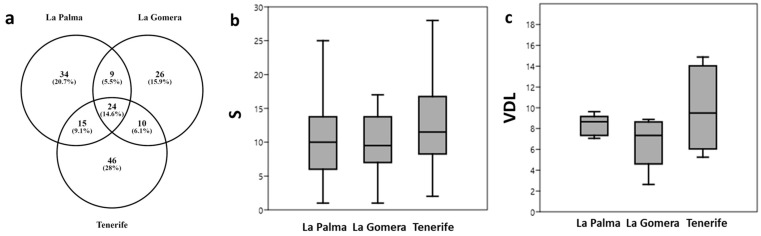
(**a**) Venn diagram of shared and exclusive lichen species by island. (**b**) Boxplot of richness (S) by island. (**c**) Boxplot of LDVs by studied island.

A total of 24 lichens were common to the three islands studied, representing 14.4% of the total identified species, while 15 (9.1%) were shared between La Palma and Tenerife, 11 (6.7%) between La Gomera and Tenerife, and 9 (5.5%) were common to La Palma and La Gomera. The number of exclusive lichens was 46 (27.9%) for Tenerife, 34 (20.6%) for La Palma, and 26 (15.8%) for La Gomera (Figure 2a).

We did not find significant differences in species richness (S) (Figure 2b), nor in the lichen diversity values (LDVs) among the studied islands (Figure 2c).

**Figure 3:**
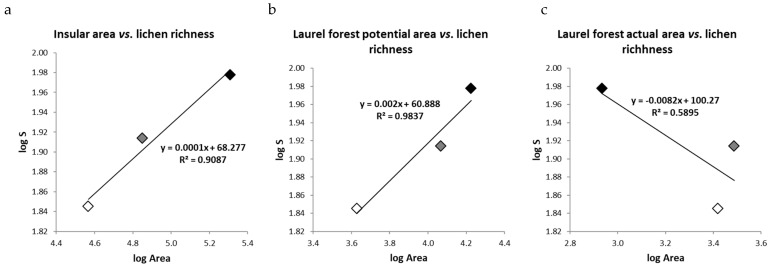
Plots log-log of linear regression of the (**a**) insular area vs. lichen richness, (**b**) humid laurel forest potential area vs. lichen richness, and (**c**) humid laurel forest actual area vs. lichen richness. Black diamond: Tenerife; grey diamond: La Palma; white diamond: La Gomera.

The size of the islands and the lichen species richness of the laurel forest presented a linear relationship. The largest island Tenerife (2034 km^2^) exhibited a higher richness than the smaller islands of La Palma (706 km^2^), and La Gomera (370 km^2^) (R^2^ = 0.97; m = 0.17) (Figure 3a). Similar results were observed when analyzing the relationship between species richness and the potential area of the laurel forest (R^2^ = 0.94; m = 0.21) (Figure 3b). However, comparing the current area of the laurel forest (Tenerife: 860 ha; La Palma: 3074 ha; La Gomera: 2621 ha) with species richness revealed an inverse relationship and a poorer fit to the line (R^2^ = 0.62; m = −0.17) (Figure 3c).

In a comprehensive island-specific analysis, we noted the predominant species within La Palma’s laurel forest to be *Parmotrema perlatum* (107 samples), *Bacidia absistens* (69), *Phlyctis agelaea* (51), *Chrysothrix candelaris* (48), and *Bacidia herbarum* (35). Similarly, the prevalent species within La Gomera’s laurel forest included *Platismatia glauca* (54 samples), *Ricasolia virens* (47), *Sticta canariensis* cyanomorphotype (39), cf. *Phyllopsora* (37), and *Leucodermia leucomelos* (31). Finally, Tenerife’s laurel forest was dominated by the following species: *Parmotrema perlatum* (75 samples), *Leucodermia leucomelos* (68), *Chrysothrix candelaris* (65), *Lepra slesvicensis* (50), and *Parmotrema robustum* (34).

### 3.2. Lichen Functional Traits

**Figure 4:**
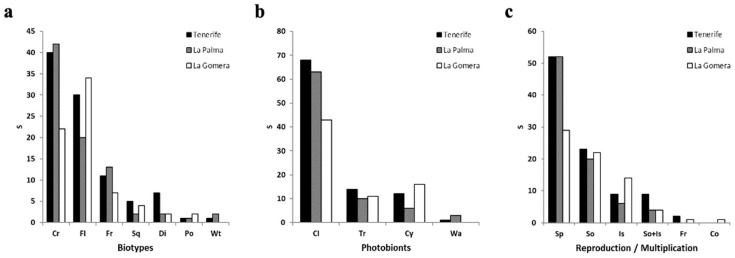
Morphological and reproductive/multiplicative. (**a**) Biotypes: Cr: crustaceous, Fl: foliaceous, Fr: fruticulous, Sq: squamulose, Di: dimorphic, Po: powdery, Wt: without thallus. (**b**) Photobionts: Cl: chlorococoid green algae, Tr: *Trentepohliaceae*, Cy: cyanobacteria, Wa: no algae. (**c**) Reproduction/multiplication: Sp: spores, So: soredia, Is: isida, So + Is: soredia and isidia, Fr: fragmentation, Co: conidia.

Examining all lichens observed on each island, we noted that the crustose (47.2%) and foliose (27.8%) types were prevalent across all islands, followed by the fruticose (12.7%) type. The lichen biota of La Gomera exhibited a different distribution, with foliose lichens (34 species) outnumbering crustose lichens (22 species) (Figure 4a). The predominant photobiont in the three islands was the chlorococcoid green algae (66.6%), followed by *Trentepohliaceae* (16.9%) and cyanobacteria (13.9%), which were present in nearly equal proportions. However, La Gomera exhibited a higher incidence of lichens with cyanobacteria (32.8%) as photobionts compared to the other two islands (7.3% and 12.6% in La Palma and Tenerife, respectively) (Figure 4b).

Regarding reproductive strategies, sexual reproduction was most prevalent in Tenerife (54.7%) and La Palma (63.4%), followed by multiplication via soredia (24.2% and 24.4%, respectively), and isidia plus soredia (9.4% and 4.8%, respectively). In La Gomera, however, the species reproducing sexually (40%) were nearly equal in number to those reproducing via soredia (31.4%). Nonetheless, sexual reproduction remained the dominant method across the epiphytic lichen diversity of the Canary laurel forest. La Gomera exhibited greater diversity in terms of reproductive mechanisms (Figure 4c).

**Figure 5:**
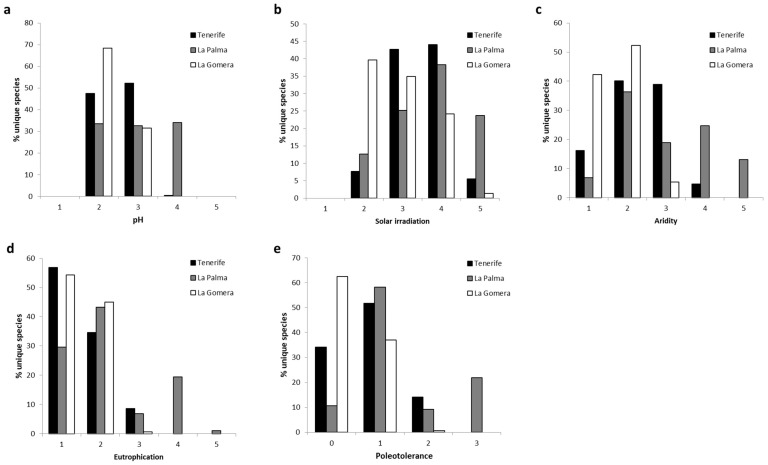
Exclusive species of the humid laurel forests of La Palma, La Gomera, and Tenerife, weighted by their frequency of occurrence in each and the highest category that they can tolerate for each of the analyzed environmental factors: (**a**) pH of the substrate, (**b**) solar irradiation, (**c**) aridity, (**d**) eutrophication, and (**e**) poleotolerance.

Considering the unique species discovered on each island, weighted by their frequency of occurrence on each island, it was evident that the lichens found in La Gomera and Tenerife were shared within category 2 of substrate pH (acid substrates), with a particularly high percentage in the case of La Gomera, and category 3 (subacid to subneutral substrates). The lichens found on La Palma were evenly distributed among the aforementioned categories and category 4 (slightly basic substrates) (Figure 5a).

Epiphytic lichens exhibited varying responses to solar irradiation on each island. The lichen composition of La Gomera was characteristic of shaded environments (category 2) and locations with abundant diffuse light but minimal direct solar irradiation (category 3), followed, to a lesser extent, by environments exposed to the sun with moderate solar irradiation (category 4). In contrast, the lichen composition of Tenerife was associated with categories 3 and 4, featuring lichens typical of both well-lit environments (category 5) and shaded environments (category 2). Meanwhile, the lichen composition of La Palma corresponded to that found in sun-exposed environments, albeit without extreme solar irradiation (category 4), followed, in similar proportions, by lichens from categories 3 and 5. It also included typical lichens of shaded environments, albeit to a lesser degree (category 2) (Figure 5b).

Regarding aridity, the composition of epiphytic lichens in the monteverde of La Palma exhibited the highest heterogeneity. While it peaked in category 2 (species typical of intermediate environments between hygrophytic and mesophytic), followed by categories 4 (typical of xerophytic environments but absent in extremely arid environments) and 3 (typical of mesophytic environments), it also included hygrophytic species typical of environments with a very high frequency of fog (category 1) as well as species typical of very xerophytic environments (category 5). Conversely, lichens in the monteverde of Tenerife and La Gomera showed greater sensitivity to aridity. In the case of Tenerife, species were largely divided between categories 2 and 3, with less than 20% associated with category 1 and virtually negligible representation (less than 5% of species) in xerophytic categories. Finally, lichens in La Gomera were primarily associated with category 2, with over 50% of species richness in this category, followed by category 1, which represented only 5.4% of species richness (Figure 5c).

Regarding eutrophication, the lichen composition of La Palma displayed notable heterogeneity, encompassing species from all categories. However, it was primarily characterized by species in category 2 (typical of environments with minimal eutrophication), followed by those in category 1 (typical of non-eutrophicated environments), and, to a lesser extent, category 4 (typical of environments with relatively high eutrophication). In contrast, the lichen composition of Tenerife and La Gomera exhibited the maximum species richness in category 1, followed by category 2, with only Tenerife displaying a small proportion of lichens assigned to category 3 (native species in low-eutrophication environments) (Figure 5d). In the monteverde of La Palma, greater heterogeneity in poleotolerance was also evident. The highest occurrence was in category 1 (species that thrive in natural and semi-natural habitats), followed by category 3 (lichens associated with highly altered environments). However, it also included species from category 0 (exclusive to old trees in ancient and intact forests) and category 2 (species that tolerate disturbed environments). Lichens in Tenerife predominantly belonged to category 1, followed by category 0, with a smaller proportion (<15% of species richness) associated with altered environments (category 2). Meanwhile, over 60% of epiphytic lichens in the monteverde of La Gomera were linked to category 0 of poleotolerance, with less than 40% associated with category 1 (Figure 5e).

### 3.3. Lichen Composition

The analysis of similarity (ANOSIM) revealed very low R values in all cases, and therefore not significant [47]. Consequently, the lichen compositions of the three islands cannot be regarded as entirely independent. It can only be affirmed that the differences in composition observed were low or practically negligible. This was evidenced by the highest R value obtained for the comparison between La Palma and La Gomera (R = 0.35), followed by that for La Palma and Tenerife (R = 0.22), and finally by the R value for the comparison between La Gomera and Tenerife (R = 0.21) (Table 3).

No significant differences were observed between the two orientations (N and S) sampled in each tree.

The SIMPER analysis revealed the highest dissimilarity value (97%) when comparing the laurel forests of La Palma vs. La Gomera. The lowest dissimilarity value (93%) was obtained when comparing the laurel forest of La Palma vs. Tenerife.

Additionally, the SIMPER analysis identified the discriminating species of the laurel forest on each island. Table 4 presents three lists of discriminating species, with the percentages of contribution of each species shown in parentheses.

Two discriminating species are shared among the three islands (*Parmotrema perlatum* and *Chrysothrix candelaris*), two are common to La Gomera and Tenerife (*Ricasolia virens* and *Leucodermia leucomelos*), and only one is common to La Palma and Tenerife (*Phlyctis agelaea*).

**Figure 6:**
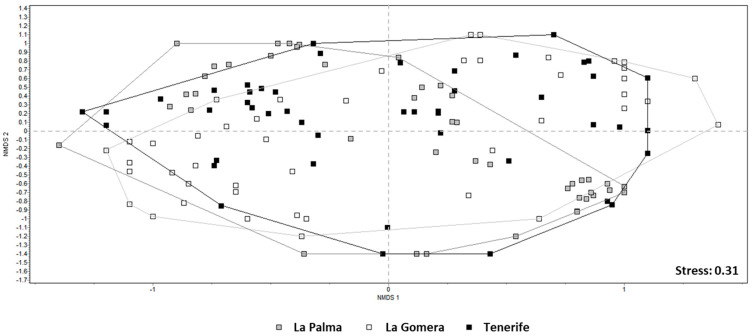
The NMDS results (Jaccard distance and abundance matrix were used). Each point represents the sum of lichen abundance in each prospected tree in each plot on the La Palma, La Gomera, and Tenerife Islands.

The NMDS ordination, using the Jaccard distance, resulted in a two-dimensional pattern with a stress value of 0.31, indicating no discernible separation among the studied islands (Figure 6).

## 4. Discussion

### 4.1. Lichen Diversity

Two primary characteristic components of lichen populations on (sub)tropical oceanic islands were identified by [48]: a pan(sub)tropical element inhabiting humid mountainous regions, characterized by a scarcity of endemic species, and a dry or semi-desert Mediterranean element exhibiting a higher degree of endemism. Their findings are consistent with the conclusions regarding endemism drawn from studies of the laurel forests of the Canary Islands, wherein only three species (*Lobaria immixta*, *Lobaria macaronesica*, and *Thelotrema laurisilvae*) were recognized as Macaronesian endemics. No endemic species from the Canary Islands were found. In contrast to the endemism percentages observed in vascular flora or invertebrate fauna, which typically range between 25% and 50%, the proportion of lichen endemism is notably lower, estimated at 2% in the Canary Islands and between 2% and 3% for the remaining Macaronesian archipelagos (Pérez-Vargas, unpublished).

Only the three species mentioned earlier are confined to the Macaronesian region [49,50,51,52]. The majority of the most common species identified in the laurel forests exhibit a broad distribution across Europe (e.g., *Ricasolia virens* and *Bacidia absistens*), while a smaller subset of species has a much broader global distribution (e.g., *Parmotrema perlatum* and *Leucodermia leucomelos*).

Some of the most frequently observed species have been previously identified as characteristic species of Canarian laurel forests [22,53,54,55]. These species are typically associated with mature forests in undisturbed or scarcely disturbed areas. In a comprehensive study of the laurel forest within Garajonay National Park (La Gomera) [56], the authors identified various epiphytic lichen communities inhabiting the tree trunks and canopy bases. Notably, communities such as *Lobarietum*, *Pertusarietum*, *Parmelietum*, and *Lecanoretum* were found, along with *Nephrometum* and *Pannarietum* in shaded areas, *Usneetum* and *Teloschistetum* in sunnier environments, and *Ramalinetum* in warmer areas. These authors also noted the presence of *Graphidetum* and *Opegraphetum* communities on the oldest trunk sections lacking bark as well as *Cladonietum* and *Peltigeretum* communities at the trunk bases, often accompanied by *Sphaerophorus globosus* and occasionally *Stereocauletum*. In this study, we observed elements from all of these previously mentioned communities.

The greater number of species found in the laurel forest of Tenerife compared to that of La Palma, and of these compared to that of La Gomera, is consistent with the theory of [9] on island biogeography, which posits a linear relationship between species richness and island size. Moreover, the unique climatic conditions present in Canarian laurel forests suggest that this habitat can be considered as an island within each island. Considering this distinctive feature, and following MacArthur and Wilson’s theory, we examined the relationship between the observed lichen diversity with the current and potential areas of this forest. The initial two relationships investigated (insular area vs. species richness and potential laurel forest area vs. species richness) exhibited a strong fit to the line (0.97 and 0.94, respectively). The slope of the line derived from island area considerations (m = 0.17) aligned with the values associated by MacArthur and Wilson with non-isolated environments on continents or within large islands (0.12 ≤ m ≤ 0.17). Conversely, the slope value of the line derived from the potential laurel forest area considerations (m = 0.21) coincided with the values these authors generally attribute to islands (0.20 ≤ m ≤ 0.35). The dimensions of the islands and the size of the remaining habitat fragments have previously been examined in other studies on island biodiversity, serving as key predictors, for instance, in explaining the current diversity of native spiders in the Azores archipelago, although this trend has not been observed in native Canarian spiders [13]. Conversely, the linear adjustment deteriorated notably when comparing the richness observed with the present extent of the laurel forest. For instance, in Tenerife, the island with the smallest proportion of its current area occupied by this forest, the observed high diversity did not align with the anticipated levels. This discrepancy prompts consideration that the epiphytic lichen biota of the Canary Islands’ laurel forest, particularly on islands as significantly altered as Tenerife, might be experiencing a phenomenon known as extinction debt [4].

This phenomenon has been investigated and verified in other organisms inhabiting this native Macaronesian forest [57]. Berglund and Jonsson [58] documented this occurrence in lichens residing in other forest ecosystems in Northern Europe. Similarly, [59] indicated that lichen populations in Mediterranean forests did not promptly respond to biodiversity losses resulting from changes in their habitat configuration, requiring an extended period for noticeable reactions to manifest. This trend may potentially be unfolding in the Macaronesian laurel forest, supported, among other studies, by the observations made in [60] on the invertebrate fauna in the Madeira archipelago.

In accordance with MacArthur and Wilson’s island biogeography theory, a shorter distance from the island to the mainland typically correlates with greater species diversity [9]. However, this hypothesis does not seem to hold true in our context. In La Gomera’s laurel forest, we observed a smaller number of species compared to La Palma, despite La Palma being farther from the presumed source (considering the “source” as both the nearest continent and the island of Tenerife, the largest of the islands under study). Consequently, we expected a lower number of shared species between Tenerife and La Palma than between Tenerife and La Gomera, and between La Palma and La Gomera. This observation could be attributed to an antagonistic relationship between island size or the area occupied by the humid laurel forest within it, and the distance to the mainland [21]. However, it could also be influenced by the varying conservation status of the Canarian laurel forests across each island.

### 4.2. Lichen Functional Traits

Lichens have been widely used as bioindicators of the environmental conditions in the areas where they inhabit [61,62]. Therefore, their functional traits have been employed, among other purposes, to characterize forest environments in terms of their conservation status [63,64,65].

In terms of epiphytic lichen biotypes, a prevailing pattern has been previously observed in forest environments, particularly in laurel forests, characterized by the dominance of crustose forms over other biotypes [24,26]. However, in the laurel forest of La Gomera, we observed a relatively different trend, with foliose species predominating over crustose ones. This observation was initially highlighted by [66] in the Garajonay National Park (La Gomera), encompassing various lichen types including terrestrial, mossy, rocky, and epiphytic lichens, with the latter constituting 52% of the lichens studied. Foliose species typically correspond to intermediate-mature and mature stages of lichen colonization, whereas crustose forms are associated with earlier stages [67,68,69]. Although some studies have reported a high diversity of crustose lichens on old trees [70], a higher prevalence of this biotype is associated with habitats characterized by significant rainfall [71]. Certain crustose species, such as those belonging to the genus *Thelotrema*, have been proposed as indicators of ecological continuity, as suggested by [72]. In the Canary Islands, three species of this genus have been identified. First, *T. lepadinum*, a pantropical element [73], has been reported for Tenerife and La Gomera, being the most frequently found species in both islands. *Thelotrema macrosporum* was recently documented for the first time in Tenerife, specifically from the Anaga Peninsula [74]. Finally, *T. laurisilvae*, has been described from Madeira and later mentioned by [75] for the island of La Palma, although it was not found in our surveys.

The higher prevalence of species displaying a dimorphic thallus in Tenerife (7.4%) is noteworthy when compared to the frequencies observed in La Gomera (2.8%) and La Palma (2.4%). All lichens exhibiting this thallus morphology belonged to the genus *Cladonia*. Some of these species have been associated with well-preserved environments within the Canarian laurel forest [66], while others have been linked to areas within this forest characterized by high levels of rainfall and humidity [53]. This is consistent with the observations made in [76] regarding the preference of *Cladoniaceae* taxa to thrive on tree trunks located in shaded environments.

Regarding the photobiont, the three islands exhibited a consistent pattern where the chlorococoid green photobiont predominated, followed by *Trentepohliaceae* and cyanolichens, as observed in recent studies conducted in the laurel forest of Tenerife [24]. Notably, La Gomera stood out for its high percentage of cyanolichens (22.5%) compared to Tenerife (12.6%) and particularly La Palma (7.3%). This percentage exceeded that reported by [66] in the Garajonay National Park (11.4%), attributed to the focus solely on epiphytes within the humid laurel forest excluding other forest environments (such as saxicolous and terrestrial habitats). Nevertheless, our percentage remained lower than that reported by [53] in the same national park, reaching 28%. This disparity could be attributed to Mester’s study primarily concentrating on macrolichens, while crustose lichens were largely overlooked.

Although recent studies have associated certain cyanolichens with arid environments [77], the requirement for water in a liquid state has conventionally been deemed necessary for their growth [78]. Despite La Palma experiencing the highest rainfall levels among the Canary Islands [79], it exhibited a lower abundance and proportion of cyanolichen species. Hence, there must be additional factors influencing their development that negatively impacts their occurrence on La Palma. Cyanolichens have also been linked to well-conserved environments and ancient forests, serving as key indicators for assessing naturalness, age, conservation status, and forest continuity [80]. Considering this, it can be inferred that the laurel forest of La Gomera demonstrates a superior conservation status compared to those of Tenerife and La Palma.

In terms of reproductive and propagative strategies, Tenerife and La Palma conformed to the overarching trend outlined in previous studies [26] where sexual reproduction predominated in the laurel forests of the Canaries. However, La Gomera stood out for its relatively low proportion of lichens exhibiting sexual reproduction (40.8%) compared to Tenerife and La Palma (54.7% and 63.4%, respectively). This reduced occurrence of sexual reproduction in La Gomera may be linked to the lower abundance of crustose lichen species, as over half of the species exhibiting this biotype engaged in sexual reproduction, along with nearly half of the lichens featuring chlorococoid green algae, which were crustose (49), distinct from the foliose (29) and fruticose (21) biotypes.

The behavior of epiphytic lichens within the laurel forest, unique to each island and evaluated based on their frequency across various environmental parameters (pH, solar irradiation, aridity, eutrophication, and shade tolerance), differed among the three islands.

Thus, regarding the pH of the substrate, La Palma stood out for its even distribution of lichens across three categories (2, 3, and 4), whereas in Tenerife and La Gomera, lichens were predominantly associated with categories 2 and 3. Particularly noteworthy was the highest occurrence of lichens exhibiting an affinity for category 2 (acidic substrates), exclusively observed in La Gomera. Mester [53] analyzed several environmental factors including lichen affinity to humidity, light, and substrate pH within the Garajonay National Park. Regarding substrate pH, Mester noted that a majority of the species within the National Park inhabited acidic substrates, consistent with our findings.

In relation to solar irradiation and aridity, the lichens recorded exclusively on La Palma are commonly found in environments characterized by higher solar irradiation and greater aridity compared to those on Tenerife and La Gomera, where they are predominantly associated with shaded and humid habitats. Mester also discussed the interplay between these two environmental factors when comparing humid laurel forests (shaded and humid environments) with *Morella faya* forests at higher altitudes (environments more exposed to high solar irradiation and aridity) [53]. The author further elucidated the vertical distribution of lichen composition based on humidity levels. Consequently, lichens resilient to desiccation dominated the canopy, whereas species thriving in shaded and humid conditions prevailed in less exposed areas of the trees. The disparities observed between La Gomera and the other two islands may be attributed to lower levels of forest management in the laurel forest of La Gomera, primarily protected as a National Park since 1981. This protected area encompasses over half of the mature laurel forest area in the archipelago [81]. Reduced forest exploitation relative to Tenerife and La Palma may have contributed to the development of a more intricate forest structure on La Gomera, impeding high solar radiation penetration into the understory while fostering greater humidity retention.

Regarding eutrophication and poleotolerance, La Palma stood out for its high frequency of lichens associated with eutrophication category 4 (19.4%) and poleotolerance category 3 (21.8%), diverging from the patterns observed in Tenerife and La Gomera, where the majority of lichens fell into the first two categories for both eutrophication and poleotolerance. Notably, La Gomera exhibited a high percentage of exclusive species typical of category 0, suggesting the favorable conservation status of its laurel forest. This observation aligns with our earlier discussion, attributing the preservation of La Gomera’s laurel forest to its designation as a National Park, despite considerable tourist activity, which has averaged almost 900,000 annual visitors in recent years [82]. These findings corroborate previous reports by other researchers [21,81,83,84] highlighting the high conservation level of La Gomera’s laurel forest, particularly within the Garajonay National Park. However, it is worth noting that the elevated eutrophication values in category 4 and poleotolerance values in category 3 on La Palma may have been influenced by the prevalence of two highly abundant species, and the inflated eutrophication and poleotolerance values were primarily attributed to *Lecidella elaeochroma* (27 observations) and *Amandinea punctata* (13 observations), which may have skewed the analysis.

### 4.3. Lichen Composition

Several studies have compared the cryptogamic biodiversity across islands [85,86,87] as well as the biodiversity within different habitats on the same island [88,89,90,91], while others have examined the biodiversity between islands and continents [92,93]. However, research that has compared the relationship between species within the same habitat on neighboring islands is scarce. Furthermore, most studies in this field have focused on genetic diversity within species of biogeographic or conservation significance, primarily concentrating on vascular plants [94,95,96].

The Canarian laurel forest has been perceived as a homogeneous habitat, despite the fact that not all species are uniformly distributed across all islands, as observed in other groups of organisms [96,97,98,99]. There is a dearth of studies examining the quality or fragmentation of Canary laurel forests using lichens as a model organism. From a conservation perspective, it is imperative to assess both the uniformity and quality of habitats present on the islands. Should variation exist among them, their management should be tailored to suit each island’s specific characteristics, particularly with regard to the biological groups that demonstrate these differences.

The ANOSIM analysis showed the absence of significant differences in the epiphytic lichen composition among the three islands studied. Furthermore, the SIMPER analysis identified the shared species predominantly contributing to elucidating the lichen composition across all three islands.

According to [47], a stress value exceeding 0.3 in the NMDS analysis, as in the case here, suggests that the data points are nearly randomly distributed in the two-dimensional ordination space, warranting caution in their interpretation. The scattered distribution of samples across the three islands, coupled with substantial overlap among them, mirrored the findings from other analyses, particularly ANOSIM. However, no evidence supports the potential differentiation of epiphytic lichen compositions among the three islands examined.

There were discriminant species shared among the studied islands. However, no lichen that served as a discriminant for a particular phorophyte was shared by more than one host species. No characteristic species of phorophyte was common to all of them. Therefore, we can affirm that there is more uniformity among the islands studied than among the four analyzed phorophytes [26].

Taking into account previous studies [56], all of the discriminating taxa of La Gomera, except cf. *Phyllopsora*, coincided with common and frequent species and were even locally abundant in Garajonay National Park.

In a study conducted in the Aguagarcía Forest (corresponding to our plot no. 4), all species identified in this study as being indicative of Tenerife were found, except for *Parmotrema robustum* and *Lepra slesvicensis* (whose presence we confirmed at this location in our current study) [54]. Among the six species identified as indicative of La Palma, *Parmotrema perlatum* and *Phlyctis agelaea* were previously reported as common by [100] in the “El Canal y Los Tiles Biosphere Reserve”. Although their study did not specifically target macrolichens, a noticeable bias toward them was evident in the species list.

Follmann [22] identified several lichen alliances and associations as the most characteristic among the corticolous species of the humid montane zone of the Canary Islands: *Xanthorion parietinae* (*Physcietum ascendentis*), *Buellion canescentis* (*Ramalinetum subgeniculatae*), *Parmelion saxatilis* (*Pseudevernietum furfuraceae*), *Lobarion pulmonariae* (*Pannarietum leucostictae*, *Nephrometum laevigatae*, and *Lobarietum meridionalis*), and *Usneion florido-ceratinae* (*Usneetum decorae*, *Usneetum rubicundae*, and *Usneetum atlanticae*). Among them, the association *Lobarietum meridionalis* stands out, found exclusively in the laurel forests of La Gomera, La Palma, and Tenerife, as the most characteristic of laurel forests, comprising species such as *Lobaria macaronesica* (as *Lobaria meridionalis*), *Crocodia aurata* (as *Pseudocyphellaria aurata*), *Leptogium cochleatum* (as *Leptogium azureum*), *Scytinium palmatum* (as *Leptogium palmatum*), *Lobaria immixta*, *Ricasolia variegata* (as *Lobaria variegata*), *Sticta canariensis* cyanomorphotype (as *Sticta dufourii*), *Sticta canariensis*, *Sticulosi weigelia*, and many other lobulate species of various sizes. On the other hand, the associations *Nephrometum laevigatae* and *Pannarietum leucostictae* are notable at the forest edges, the former characterizing sites with the highest incidence of light, and the latter, locations with the highest rainfall.

Furthermore, *Scytinium palmatum* is cited for all three islands, and while its distribution is closely associated with the laurel forest area, it is not as common as other characteristic species found in this environment. For instance, in Garajonay National Park, [56] only sporadically found it, typically on wet slopes with bryophytes rather than as epiphytes. Intriguingly, this same species has been documented in the La Caldera de Taburiente National Park [101] within the Canarian pine forest area, where its presence is linked to wet mossy slopes, albeit it remains rare in the National Park. Moreover, the limited records of this species in Tenerife are primarily associated with laurel forests, particularly in mossy soils, and it has only been sporadically located as an epiphyte [102,103,104].

The known distribution and the issues surrounding the three epiphytic taxa (*Ricasolia variegata*, *Scytinium palmatum*, and *Sticta weigelii*) discussed above lead us to suggest that they should not be regarded as an indicator species of the Canary laurel forest, considering the abundance of other viable candidates. For instance, species like *Cladonia squamosa*, *Heterodermia obscurata*, *Hypogymnia tavaresii*, and *Pyrenula occidentalis* could be utilized for this purpose. Furthermore, genera such as *Lobaria*, *Micarea*, *Phlyctis*, *Pseudocyphellaria* (including *Crocodia*), *Sticta*, and *Thelotrema* appear to be more prevalent and specific to laurel forests compared to the aforementioned three species.

From a lichenological perspective, the most studied localities in monteverde for the island of La Palma have been within the Biosphere Reserve of El Canal y Los Tilos [100] and in Garajonay in La Gomera [56]. All species mentioned in these previous studies have been located in our surveys in the monteverde of the three islands studied, with no significant differences observed among them. These results support the homogeneity in terms of the lichen biota composition of the Canarian laurel forest.

Boch et al. [105] called for the strict protection of Madeira laurel forests in order to minimize anthropic damage and develop management measures aimed at habitat improvement and the implementation of species conservation programs limiting future extinctions, especially those that were endemic. The existence of a possible extinction debt and the characteristics of each of the habitats when we analyzed the functional traits of the characteristic lichen species, leads us to propose, like Boch for Madeira, that conservation measures be implemented to protect the lichens of this forest in the Canarian archipelago.

## 5. Conclusions

The epiphytic lichen biodiversity of the Canarian humid laurel forest is characterized by a low rate of endemism when compared to other taxonomic groups such as vascular plants or arthropods, alongside a high number of species shared between at least two islands. Its uniformity in terms of lichen composition might suggest that the Canarian laurel forest is similarly conserved across the three islands under study. However, we have uncovered evidence of an extinction debt on the island of Tenerife, likely stemming from the decline of this habitat in recent centuries. Furthermore, evidence indicating varying degrees of conservation has been detected. In this regard, La Gomera stands out for its higher degree of conservation of laurel forests compared to that of Tenerife and La Palma.

## Figures and Tables

**Table 1:** Field plots.

Plot	Island	Locality	UTM	Altitude
1	Tenerife	Pista Las Hiedras (Anaga)	375110/3157614	830
2	Tenerife	Erjos (Teno)	321907/3134428	860
3	Tenerife	Cueva del Guanche (Anaga)	380235/3157573	840
4	Tenerife	Aguagarcía	362558/3148881	885
5	Tenerife	Hoya de Ijuana (Anaga)	385900/3159815	680
6	Tenerife	Las Portelas (Teno)	320500/3135776	840
7	La Palma	Mirador de Las Mimbreras	223103/3190532	770
8	La Palma	Don Pedro	216864/3191489	860
9	La Palma	Laguna de Barlovento	225903/3190442	750
10	La Palma	Hoyo del Infierno	226243/3168304	950
11	La Palma	Roque Niquiomo	225640/3166681	1050
12	La Palma	La Pavona	225015/3170197	825
13	La Gomera	Lomito Ventoso	273628/3116431	995
14	La Gomera	Cordilleras de Vallehermoso	276756/3115483	1090
15	La Gomera	El Bailadero	283088/3112836	1010
16	La Gomera	El Contadero	279720/3112282	1000
17	La Gomera	Fuensanta	278779/3115994	995
18	La Gomera	La Gollada Colorada	278663/3114210	1160

**Table 2:** Functional traits: ecological requirement. Extracted from [37].

Functional Traits	Value	Signification
Tolerance to eutrophication	1	Lichens not resistant to eutrophication
	2	Lichens resistant to a very weak eutrophication
	3	Lichen resistant to a weak eutrophication
	4	Lichen occurring in rather eutrophicated situations
	5	Lichens occurring in highly eutrophicated situations
Water requirements	1	Hydro and hygrophytic species, in sites with a very high frequency of fog
	2	Rather hygrophytic species, intermediate between 1 and 3
	3	Mesophytic species
	4	Xerophytic species, but absent from extremely and stands
	5	Very xerophytic species
Solar irradiation	1	Species growing in very shaded situations
	2	Species growing in shaded situation
	3	Species growing in sites with plenty of diffuse light but scarce direct solar irradiation
	4	Species growing in sun-exposed sited, but avoiding extreme solar irradiation
	5	Species growing in sites with very high direct solar irradiation
Poleotolerance	0	Lichens which exclusively occur on old trees in ancient, undisturbed forests
	1	Lichens mostly occurring in natural or semi-natural habitats
	2	Lichens occurring also in moderately disturbed areas (agricultural areas, small settlements, etc.)
	3	Lichens occurring also in heavily disturbed areas, incl. large towns
pH of the substrata	1	Species which occur on very acid substrata
	2	Species which occur on acid substrata
	3	Species which occur on subacid to subneutral substrata
	4	Species which occur on slightly basic substrata
	5	Species which occur on basic substrata

**Table 3:** The ANOSIM and SIMPER results. The ANOSIM values are in the upper of the diagonal, and the SIMPER values are on and under the diagonal. (*: *p* = 10^−3^).

	La Palma	La Gomera	Tenerife
La Palma	12%	0.35 *	0.22 *
La Gomera	97%	9.4%	0.21 *
Tenerife	93%	95%	11%

**Table 4:** The SIMPER results. Discriminant lichen species of La Palma, La Gomera, and Tenerife humid laurel forests. We show all of the species that collectively contribute to 75% of the total on each island. Percentages of contribution of each species are shows in brackets.

La Palma	La Gomera	Tenerife
*Parmotrema perlatum* (37%) *Bacidia absistens* (13%) *Phlyctis agelaea* (11%) *Byssoloma subdiscordans* (6.1%) *Chrysothrix candelaris* (4.3%)	*Platismatia glauca* (15.1%) *Ricasolia virens* (13.1%) *Thelotrema lepadinum* (10.1%) cf. *Phyllopsora* (8.5%) *Parmotrema crinitum* (7.2%) *Sticta canariensis* cyanomorph (5.7%) *Leucodermia leucomelos* (4.8%) *Normandina pulchella* (3.6%) *Chrysothrix candelaris* (3.6%) *Parmotrema perlatum* (3.3%)	*Parmotrema perlatum* (23%) *Pertusaria slesvicensis* (14%) *Leucodermia leucomelos* (12%) *Chrysothrix candelaris* (8.6%) *Ochrolechia pallescens* (3.8%) *Phlyctis agelaea* (3.3%) *Parmotrema robustum* (3.2%) *Parmotrema reticulatum* (2.5%) *Teloschistes flavicans* (2.4%) *Ricasolia virens* (2.2%)

## Data Availability

Data are contained within the article.

## Supplementary Materials

The following supporting information can be downloaded at: https://www.mdpi.com/article/10.3390/jof10100668/s1, Table S1: Species identified in the present work. The frequency of occurrence on each island is shown as well as the studied functional traits values. Abbreviations: Biotypes: Cr: crustaceous, Fl: foliaceous, Fr: fruticulous, Sq: squamous, Di: dimorphic, Po: powdery, Wt: without thallus; photobionts: Cl: chlorococoid green algae, Tr: *Trentepohliaceae*, Cy: cyanobacteria, Wa: no algae; and reproduction/multiplication: Sp: spores, So: soredia, Is: isidia, So + Is: soredia and isidia, Fr: fragmentation, Co: conidia. *: This symbol indicates that the values for pH, solar irradiation, aridity, eutrophication, and poleotolerance were assigned according to the criteria of expert Canarian lichenologists and our own field observations.
