# Supplementary material for: On the Conservation of the Canarian Laurel Forest: What Do Lichens Have to Say?
Source: J Fungi (Basel). 2024 Sep 25;10(10):668. doi: 10.3390/jof10100668 (PMC11508214; doi:10.3390/jof10100668)
Supplement: Supplementary file 1 [file jof-10-00668-s001.zip › jof-3071441-supplementary.pdf]

Supplementary Material.

Table S1. Species identified in the present work. The frequency of occurrence on each island is shown, as well as the studied functional traits values. Abbreviations: Biotypes: Cr: crustose, Fl: foliaceous, Fr: fruticulous, Sq: squamous, Di: dimorphic, Po: powdery, Wt: without thallus; photobionts: Cl: chlorococoid green algae, Tr: *Trentepohliaceae*, Cy: cyanobacteria, Wa: no algae; and reproduction / multiplication: Sp: spores, So: soredia, Is: isidia, So + Is: soredia and isidia, Fr: fragmentation, Co: conidia. *: This symbol indicates that the values for pH, solar irradiation, aridity, eutrophication and poleotolerance were assigned according to the criteria of expert Canarian lichenologists and our own field observations.

**Species**	**Tenerife**	**La Palma**	**La Gomera**	**Biotype**	**Fotobiont**	**Reproduction / Multiplication**	**pH**	**SI**	**Ar**	**Eu**	**PI**	*
Acrocordia gemmata (Ach.) A. Massal.	0	0	1	Cr	Tr	Sp	3	3	2	1	1	
Alectoria imshaugii Brodo & D. Hawksw.	0	0	1	Fr	Cl	Is	2	3	2	1	0	*
Alyxoria culmigena (Lib.) Ertz	13	1	0	Cr	Tr	Sp	3	3	3	2	1	
Alyxoria ochrocincta (Werner) Ertz	0	1	0	Cr	Tr	Sp	3	4	2	2	1	
Alyxoria varia (Pers.) Ertz & Tehler	0	5	0	Cr	Tr	Sp	3	3	3	2	2	
Amandinea punctata (Hoffm.) Coppins & Scheid.	0	13	0	Cr	Cl	Sp	3	5	4	4	3	
Anisomeridium polypori (Ellis & Everh.) M.E. Barr	0	8	0	Cr	Tr	Sp	4	2	2	2	1	
Anisomeridium ranunculosporum (Coppins & P. James) Coppins	0	2	0	Cr	Tr	Sp	3	4	3	1	2	
Arthonia ilicina Taylor	0	0	1	Cr	Tr	Sp	3	3	2	2	0	
***Arthonia* sp. 1**	7	0	0	Cr	Tr	Sp	2	2	2	2	1	
Arthonia stellaris Kremp.	0	1	0	Cr	Tr	Sp	2	3	2	1	0	
Athallia holocarpa (Hoffm.) Arup, Frödén & Søchting	4	28	0	Cr	Tr	Sp	3	5	5	5	3	
Bacidia absistens (Nyl.) Arnold	0	69	4	Cr	Cl	Sp	3	3	2	1	1	
Bacidia arceutina (Ach.) Arnold	0	2	0	Cr	Cl	Sp	3	4	3	2	1	
Bacidia herbarum (Stizenb.) Arnold	0	35	0	Cr	Cl	Sp	4	4	4	2	1	
Bacidia laurocerasi (Delise *ex* Duby) Zahlbr.	1	21	0	Cr	Cl	Sp	2	4	2	1	0	
Bacidia rosella (Pers.) De Not.	0	1	0	Cr	Cl	Sp	3	3	2	3	0	
Bacidia rubella (Hoffm.) A. Massal.	0	5	0	Cr	Cl	Sp	3	4	3	3	2	
Bibbya vermifera (Nyl.) Kistenich, Timdal, Bendiksby & S. Ekman	0	1	0	Cr	Cl	Sp	2	3	2	2	1	

Blastenia ferruginea (Huds.) A. Massal.	2	8	0	Cr	Tr	Sp	3	5	3	3	2	
Bryoria chalybeiformis (L.) Brodo & D. Hawksw.	0	1	0	Fr	Cl	So	2	5	3	3	1	
Bryoria furcellata (Fr.) Brodo & D. Hawksw.	0	0	2	Fr	Cl	Is+So	2	5	3	1	1	
Bryoria fuscescens (Gyeln.) Brodo & D. Hawksw.	0	2	2	Fr	Cl	So	3	5	2	2	1	
Buellia disciformis (Fr.) Mudd	14	3	0	Cr	Cl	Sp	2	4	2	2	1	
Buellia leptoclinoides (Nyl.) J. Steiner	1	0	0	Cr	Cl	Sp	3	4	2	1	1	
Byssoloma marginatum (Arnold) Sérus.	0	2	0	Cr	Cl	Sp	2	3	1	1	0	
Byssoloma subdiscordans (Nyl.) P. James	0	33	4	Cr	Cl	Sp	2	3	1	1	0	
Calicium glaucellum Ach.	0	2	0	Wt	Wa	Sp	2	3	2	1	0	
Chrysothrix candelaris (L.) J.R. Laundon	65	48	29	Po	Cl	So	2	3	3	1	2	
Cladonia carneola (Fr.) Fr.	2	5	0	Di	Cl	So	2	4	3	1	1	
Cladonia coniocraea (Flörke) Spreng.	9	5	0	Di	Cl	So	3	4	3	3	2	
Cladonia fimbriata (L.) Fr.	0	0	1	Di	Cl	So	3	4	3	3	2	
Cladonia floerkeana (Fr.) Flörke	1	0	0	Di	Cl	Sp	2	4	3	1	1	
Cladonia macilenta Hoffm.	1	0	0	Di	Cl	So	2	4	3	2	2	
Cladonia merochlorophaea Asahina	0	0	5	Di	Cl	So	2	4	3	1	1	
Cladonia parasitica (Hoffm.) Hoffm.	16	0	0	Di	Cl	Fr	2	3	3	1	1	
Cladonia squamosa (Scop.) Hoffm.	2	0	0	Di	Cl	Fr	2	3	3	2	1	
Cladonia subulata (L.) Weber *ex* F.H. Wigg.	1	0	0	Di	Cl	So	3	4	3	1	1	
Coenogonium luteum (Dicks.) Kalb & Lücking	2	1	3	Cr	Tr	Sp	2	2	2	2	0	
Coenogonium pineti (Ach.) Lücking & Lumbsch	0	4	1	Cr	Tr	Sp	3	3	3	2	2	
Collema subnigrescens Degel.	4	0	0	Fl	Cy	Sp	3	4	2	3	2	
Coniocarpon cinnabarinum DC.	7	0	0	Cr	Tr	Sp	3	4	3	1	1	
Fuscopannaria mediterranea (Tav.) P.M. Jørg.	0	0	2	Sq	Cy	So	3	4	2	2	1	
Graphis scripta (L.) Ach.	1	0	0	Cr	Tr	Sp	3	3	3	2	2	
Heterodermia leucomelos (L.) Poelt	68	18	31	Fl	Cl	So	3	4	1	2	0	*
Heterodermia obscurata (Nyl.) Trevis.	19	0	1	Fl	Cl	So	3	4	2	3	2	
Hypogymnia physodes (L.) Nyl.	1	0	2	Fl	Cl	So	3	4	3	2	3	
Hypogymnia tubulosa (Schaer.) Hav.	1	0	0	Fl	Cl	So	2	3	3	2	2	
Hypotrachyna endochlora (Leight.) Hale	6	0	6	Fl	Cl	So	2	3	2	2	1	*

Hypotrachyna laevigata (Sm.) Hale	2	1	13	Fl	Cl	So	2	3	2	1	0	
Hypotrachyna minarum (Vain.) Krog & Swinscow	5	6	0	Fl	Cl	Is	2	3	2	1	0	
Hypotrachyna revoluta (Flörke) Hale	5	3	2	Fl	Cl	So	2	3	3	3	2	
Hypotrachyna rockii (Zahlbr.) Hale	2	0	0	Fl	Cl	So	2	3	3	3	2	*
Hypotrachyna sinuosa (Sm.) Hale	0	3	0	Fl	Cl	So	2	3	2	1	0	
Hypotrachyna taylorensis (M.E. Mitch.) Hale	1	0	0	Fl	Cl	Sp	2	3	2	1	0	
Lecanactis abietina (Ach.) Körb.	0	0	5	Cr	Tr	Sp	2	2	2	1	0	
Lecanora albella (Pers.) Ach.	0	18	0	Cr	Cl	Sp	2	3	2	1	1	
Lecanora allophana (Ach.) Nyl.	1	0	0	Cr	Cl	Sp	3	5	4	3	2	
Lecanora argentata (Ach.) Röhl.	3	4	0	Cr	Cl	Sp	3	4	3	2	2	
Lecanora chlarotera Nyl.	2	2	0	Cr	Cl	Sp	3	5	4	5	3	
Lecanora gangaleoides Nyl.	1	0	0	Cr	Cl	Sp	3	3	3	2	2	
Lecanora hybocarpa (Tuck.) Brodo	10	0	0	Cr	Cl	Sp	3	5	4	2	2	
Lecanora pulicaris (Pers.) Ach.	13	16	1	Cr	Cl	Sp	2	5	4	2	2	
Lecanora rubicunda Bagl.	5	28	0	Cr	Cl	Sp	3	5	3	3	1	
Lecanora rugosella Zahlbr.	0	2	0	Cr	Cl	Sp	3	5	4	5	3	
Lecanora symmicta (Ach.) Ach.	2	0	0	Cr	Cl	Sp	2	4	3	2	2	
Lecidella elaeochroma (Ach.) M. Choisy	0	27	0	Cr	Cl	Sp	4	5	5	4	3	
Lepra albescens (Huds.) Hafellner	4	0	0	Cr	Cl	Sp	3	4	3	3	2	
Lepra amara (Ach.) Hafellner	9	2	8	Cr	Cl	Sp	3	4	3	3	3	
Lepra multipuncta (Turner) Hafellner	1	0	0	Cr	Cl	Sp	2	3	2	2	1	
Lepra ophthalmiza (Nyl.) Hafellner	28	0	5	Cr	Cl	Sp	2	3	2	1	1	
Lepra slesvicensis (Erichsen) Hafellner	50	0	2	Cr	Cl	Sp	2	4	3	2	1	
Leptogium* aff. *cyanescens	0	0	3	Fl	Cy	Is	3	3	2	1	0	
Leptogium brebissonii Mont.	0	0	5	Fl	Cy	Is	3	3	2	2	1	
Leptogium cochleatum (Dicks.) P.M. Jørg. & P. James	1	1	10	Fl	Cy	Sp	3	3	2	2	0	
Leptogium coralloideum (Meyen & Flot.) Vain.	0	5	1	Fl	Cy	Is	3	3	2	2	1	
Leptogium cyanescens (Ach.) Körb.	17	0	7	Fl	Cy	Is	3	3	2	1	0	
Leptogium teretiusculum Wallr. *ex* Arnold	0	3	2	Fl	Cy	Is	3	4	3	3	2	
Leucodermia boryi (Fée) Kalb	0	0	18	Fl	Cl	So	3	4	1	2	0	*
Lobaria immixta Vain.	19	8	10	Fl	Cl	Sp	3	3	2	2	1	*

Lobaria macaronesica C. Cornejo & Scheid.	21	12	23	Fl	Cl	Is	3	3	2	2	1	*
Megalaria grossa (Pers. *ex* Nyl.) Hafellner	0	1	0	Cr	Cl	Sp	3	3	2	2	0	
Micarea alabastrites (Nyl.) Coppins	20	6	12	Cr	Cl	Sp	3	3	2	2	2	*
Micarea pycnidiophora Coppins & P. James	0	2	5	Cr	Cl	Sp	2	2	2	1	1	*
Micarea stipitata Coppins & P. James	0	9	14	Cr	Cl	Sp	3	2	2	1	1	*
Micarea synotheoides (Nyl.) Coppins	0	0	1	Cr	Cl	Sp	2	2	2	2	1	*
Mycoporum lacteum (Ach.) R.C. Harris	0	3	0	Cr	W a	Sp	3	3	3	2	1	*
Nephroma laevigatum Ach.	2	0	0	Fl	Cy	Sp	3	3	2	1	1	
Nephromopsis chlorophylla (Willd.) Divakar, A. Crespo & Lumbsch	0	1	8	Fl	Cl	So	2	4	3	2	1	
Normandina pulchella (Borrer) Nyl.	3	7	23	Sq	Cl	So	3	4	3	3	2	
Ochrolechia balcanica Verseghy	1	0	0	Cr	Cl	Sp	3	3	2	2	1	
Ochrolechia pallescens (L.) A. Massal.	32	0	0	Cr	Cl	Sp	3	4	3	2	1	
Ochrolechia szatalaensis Verseghy	2	0	0	Cr	Cl	Sp	2	3	3	1	1	
Pannaria rubiginosa (Ach.) Delise	1	0	5	Sq	Cy	Sp	3	3	2	1	0	
Parmelia saxatilis (L.) Ach.	4	6	3	Fl	Cl	Is	2	4	3	3	2	
Parmelia sulcata Taylor	0	2	0	Fl	Cl	So	3	5	3	3	3	
Parmelinopsis horrescens (Taylor) Elix & Hale	10	0	17	Fl	Cl	Is	3	3	2	2	0	
Parmotrema arnoldii (Du Rietz) Hale	0	0	8	Fl	Cl	So	2	3	2	1	0	
Parmotrema crinitum (Ach.) M. Choisy	18	0	30	Fl	Cl	Is	2	3	2	1	1	
Parmotrema perlatum (Huds.) M. Choisy	75	107	20	Fl	Cl	So	2	4	3	2	2	
Parmotrema reticulatum (Taylor) M. Choisy	29	13	6	Fl	Cl	So	3	4	2	2	2	
Parmotrema robustum (Degel.) Hale	34	0	0	Fl	Cl	So	2	4	2	1	0	
Pectenia atlantica (Degel.) P.M. Jørg., L. Lindblom, Wedin & S. Ekman	3	0	0	Sq	Cy	Is	3	3	1	1	0	
Pectenia ligulata (P.M. Jørg. & P. James) P.M. Jørg., L. Lindblom, Wedin & S. Ekman	3	0	0	Sq	Cy	Is	2	2	2	2	1	*
Pectenia plumbea (Lightf.) P.M. Jørg., L. Lindblom, Wedin & S. Ekman	13	5	0	Sq	Cy	Sp	3	3	2	2	0	
Pertusaria coccodes (Ach.) Nyl.	1	0	0	Cr	Cl	Sp	3	4	2	3	1	
Pertusaria dispar J. Steiner	1	0	0	Cr	Cl	Sp	3	4	3	3	1	

Pertusaria hymenea (Ach.) Schaer.	0	2	0	Cr	Cl	Sp	3	5	3	2	2	
Pertusaria leioplaca (Ach.) DC.	0	11	0	Cr	Cl	Sp	2	4	3	2	1	
Pertusaria werneriana Boqueras	1	0	0	Cr	Cl	Sp	3	4	3	2	1	
Phlyctis agelaea (Ach.) Flot.	31	51	2	Cr	Cl	Sp	3	3	3	2	2	
Phlyctis argena (Ach.) Flot.	4	0	0	Cr	Cl	So	2	3	3	2	2	
***Phyllopsora* cf.**	0	0	37	Fl	Cl	Fr	2	2	2	2	1	
Piccolia ochrophora (Nyl.) Hafellner	0	3	0	Cr	Cl	Sp	3	4	3	3	2	
Platismatia glauca (L.) W.L. Culb. & C.F. Culb.	4	4	54	Fl	Cl	So	2	5	3	2	2	
Polyblastidium japonicum (M. Satô) Kalb	0	0	2	Fl	Cl	So	3	3	1	2	0	*
Porina atlantica (Erichsen) P.M. Jørg.	0	0	1	Cr	Tr	Sp	2	2	1	1	0	*
Porina effilata M. Brand & Sérus.	0	0	1	Cr	Tr	Sp	2	2	1	1	0	*
Pseudocyphellaria aurata (Ach.) Vain.	26	16	4	Fl	Cl	So	3	3	1	1	0	
Pseudocyphellaria crocata (L.) Vain.	0	0	1	Fl	Cy	So	2	2	1	1	0	*
Pseudocyphellaria intricata (Delise) Vain.	0	0	11	Fl	Cy	So	3	2	1	1	0	*
Pseudosagedia aenea (Körb.) Hafellner & Kalb	2	0	0	Cr	Tr	Sp	3	2	3	1	1	
Pyrenula dermatodes (Borrer) Schaer.	0	0	1	Cr	Tr	Sp	2	2	2	2	1	*
Pyrenula occidentalis (R.C. Harris) R.C. Harris	14	0	0	Cr	Tr	Sp	2	3	2	1	1	
Pyrenula pseudobufonia (Rehm) R.C. Harris	1	0	0	Cr	Tr	Sp	2	3	2	1	1	*
Ramalina chondrina J. Steiner	0	9	0	Fr	Cl	Sp	2	3	1	1	0	*
Ramalina farinacea (L.) Ach.	4	6	0	Fr	Cl	So	3	5	2	2	2	
Ramalina fastigiata (Pers.) Ach.	0	2	0	Fr	Cl	Sp	3	5	3	3	2	
Ramalina peruviana Ach.	0	3	0	Fr	Cl	So	2	3	1	1	0	*
Ramalina pusilla Le Prévost *ex* Duby	0	3	0	Fr	Cl	Sp	3	4	2	2	1	
Ramalina subgeniculata Nyl.	0	15	0	Fr	Cl	Sp	2	4	2	2	1	
Reichlingia anombrophila (Coppins & P. James) Frisch	0	0	1	Cr	Tr	Sp	2	4	2	1	0	
Ricasolia virens (With.) H.H. Blom & Tønsberg	21	5	47	Fl	Cl	Sp	3	3	1	1	0	
Rinodina capensis Hampe	0	1	0	Cr	Cl	Sp	3	4	3	2	1	
Rinodina septentrionalis Malme	0	1	0	Cr	Cl	Sp	2	4	3	2	1	
Scytinium aragonii (Otálora) Otálora, P.M. Jørg. & Wedin	1	0	0	Fl	Cy	Sp	4	3	3	2	2	
Scytinium tenuissimum (Hoffm.) Otálora, P.M. Jørg. & Wedin	1	0	0	Fl	Cy	Sp	3	4	3	3	1	

Sphaerophorus globosus (Huds.) Vain.	0	0	9	Fr	Cl	Sp	2	4	2	1	0	
Sphinctrina leucopoda Nyl.	0	3	0	Wt	Wa	Sp	2	3	2	1	1	
Stenocybe septata (Leight.) A. Massal.	6	0	0	Wt	Wa	Sp	2	3	3	3	1	*
Sticta canariensis (Bory) Bory *ex* Delise	29	0	0	Fl	Cl	Sp	3	3	1	1	0	
Sticta ciliata Taylor	0	0	9	Fl	Cy	Is	2	3	1	1	0	*
Sticta dufourii Delise	7	1	39	Fl	Cy	Is	3	3	1	1	0	
Sticta fuliginoides Magain & Sérus.	0	0	16	Fl	Cy	Is	2	3	1	1	0	*
Sticta fuliginosa (Dicks.) Ach.	0	0	3	Fl	Cy	Is	2	3	1	1	0	
Sticta limbata (Sm.) Ach.	1	4	9	Fl	Cy	So	2	3	1	1	0	
Strigula tagananae (Harm.) R.C. Harris	6	0	0	Cr	Tr	Sp	3	2	1	1	1	*
Syncesia myrticola (Fée) Tehler	6	0	0	Cr	Tr	Sp	3	4	2	2	1	
Teloschistes flavicans (Sw.) Norman	23	3	0	Fr	Cl	So	3	5	1	3	0	
Tephromela atra (Huds.) Hafellner	0	1	0	Cr	Cl	Sp	3	4	4	2	3	
Thelotrema laurisilvae Lücking & Breuss	0	0	1	Cr	Tr	Sp	2	2	1	1	1	*
Thelotrema lepadinum (Ach.) Ach.	12	0	28	Cr	Tr	Sp	2	3	2	1	0	
Thelotrema macrosporum P.M. Jørg. & P. James	6	0	0	Cr	Tr	Sp	2	3	2	1	0	*
Usnea barbata (L.) F.H. Wigg.	1	0	0	Fr	Cl	Is+So	2	4	2	1	0	
Usnea chaetophora Stirt.	1	0	0	Fr	Cl	Is+So	2	5	3	1	1	*
Usnea cornuta Körb.	7	7	1	Fr	Cl	Is+So	2	5	2	1	1	
Usnea diplotypus Vain.	3	2	1	Fr	Cl	Is+So	2	5	3	1	1	
Usnea flammea Stirt.	3	2	0	Fr	Cl	Is+So	2	5	2	1	1	
Usnea schadenbergiana Göpp. & Stein	4	0	0	Fr	Cl	Is+So	2	4	2	1	1	*
Usnea subflammea P. Clerc	1	0	0	Fr	Cl	Is+So	2	4	2	1	1	*
Usnea subfloridana Stirt.	1	0	0	Fr	Cl	Is+So	3	5	3	2	2	
Usnea subscabrosa Nyl. *ex* Motyka	13	6	4	Fr	Cl	Is+So	2	4	2	2	1	
Vahliella saubinetii (Mont.) P.M. Jørg.	0	0	3	Sq	Cy	Sp	3	3	2	1	0	
Varicellaria velata (Turner) I. Schmitt & Lumbsch	6	0	0	Cr	Cl	Sp	3	3	2	2	0	
***Vezdaea* sp. 1**	0	18	0	Cr	Cl	Sp	3	2	2	1	1	*
